# N-methyl D-aspartate receptor subtype 2B antagonist, Ro 25-6981, attenuates neuropathic pain by inhibiting postsynaptic density 95 expression

**DOI:** 10.1038/s41598-018-26209-7

**Published:** 2018-05-18

**Authors:** Ling-Er Huang, Shao-Hui Guo, Lalita Thitiseranee, Yan Yang, Yan-Feng Zhou, Yong-Xing Yao

**Affiliations:** 1 First Affiliated Hospital, Zhejiang University School of Medicine, Department of Anesthesia, Hangzhou, 310003 P.R. China; 2 Zhejiang University School of Medicine, Hangzhou, 310016 P.R. China; 3 Zhejiang University School of Medicine, Centre for Neuroscience, Hangzhou, 310016 P.R. China

## Abstract

Postsynaptic density-95 (PSD-95) is a synaptic scaffolding protein that plays a crucial role in the development of neuropathic pain. However, the underlying mechanism remains unclear. To address the role of PSD-95 in N-methyl-D-aspartate receptor subtype 2B (NR2B) -mediated chronic pain, we investigated the relationship between PSD-95 activation and NR2B function in the spinal cord, by using a rat model of sciatic nerve chronic constriction injury (CCI). We demonstrate that the expression levels of total PSD-95 and cAMP response element binding protein (CREB), as well as phosphorylated NR2B, PSD-95, and CREB, in the spinal dorsal horn, and the interaction of NR2B with PSD-95 were increased in the CCI animals. Intrathecal injection of the selective NR2B antagonist Ro 25-6981 increased paw withdrawal latency, in a thermal pain assessment test. Moreover, repeated treatment with Ro 25-6981 markedly attenuated the thermal hypersensitivity, and inhibited the CCI-induced upregulation of PSD-95 in the spinal dorsal horn. Furthermore, intrathecal injection of the PSD-95 inhibitor strikingly reversed the thermal and mechanical hyperalgesia. Our results suggest that blocking of NR2B signaling in the spinal cord could be used as a therapeutic candidate for treating neuropathic pain.

## Introduction

Neuropathic pain, usually caused by a primary lesion in the nervous system, is a serious worldwide public health problem^1,2^. Behaviorally, it is characterized by aberrant spontaneous pain, alterations in pain perception, and stimulus-evoked abnormal pain symptoms, such as hyperalgesia and allodynia. Current therapy is often ineffective due to the poor understanding of the complex pathologic mechanisms involved. It is estimated that more than 30% of the general population is affected by persisting pain, which often becomes pathological and debilitating, and causes people to seek medical attention^3–6^.

Postsynaptic density-95 (PSD-95) protein anchors glutamatergic N-methyl-D-aspartate (NMDA) receptors (NMDAR) to intracellular signaling molecules, at the level of neuronal synapses, thus modulating the specificity of pain-related glutamatergic neurotransmission signaling cascades^7,8^. Recent studies have demonstrated the involvement of PSD-95 in neuropathic pain development, induced by peripheral nerve injury^9,10^. It has been shown that intrathecal delivery of an NMDA subtype 2B (NR2B)-mimetic peptide attenuates both neuronal hyperexcitability and abnormal pain-related behaviors by perturbing PSD-95-NR2B interactions^11,12^, suggesting that both proteins play an essential role in the generation of central sensitization. On the other hand, central sensitization is considered to contribute to the formation and development of chronic pain states^13–15^. However, the role and the underlying mechanism of the relationship between PSD-95 and NR2B in the development or maintenance of neuropathic pain are largely unknown. For example, some studies have shown that spinal NR2B expression increases upon partial constrictive injury of the sciatic nerve in rats^16^, while others have suggested that pain transmission signals are not induced by NR2B protein alterations^17–20^.

These discrepancies prompted us to further study the mechanism by which NR2B and PSD-95 mediate neuropathic pain. For this purpose, we employed the chronic constrictive injury (CCI) rat model to simulate clinical neuropathic pain, and we investigated the relationship between PSD-95 activation and NR2B function in the spinal dorsal horn, by means of intrathecal injections of selective NR2B and PSD-95 antagonists. Our aim was to characterize the role of spinal NR2B and PSD-95 in the generation and development of nerve injury-induced neuropathic pain and provide a new direction for the treatment of chronic pain. We hypothesized that CCI induces activation of spinal cord NR2B/PSD-95/cAMP response element binding protein (CREB) signaling and that perturbing NR2B function might attenuate pain hypersensitivity.

## Results

### Spinal cord expression of NR2B and co-localization with PSD-95 in normal rats

**Figure 1:**
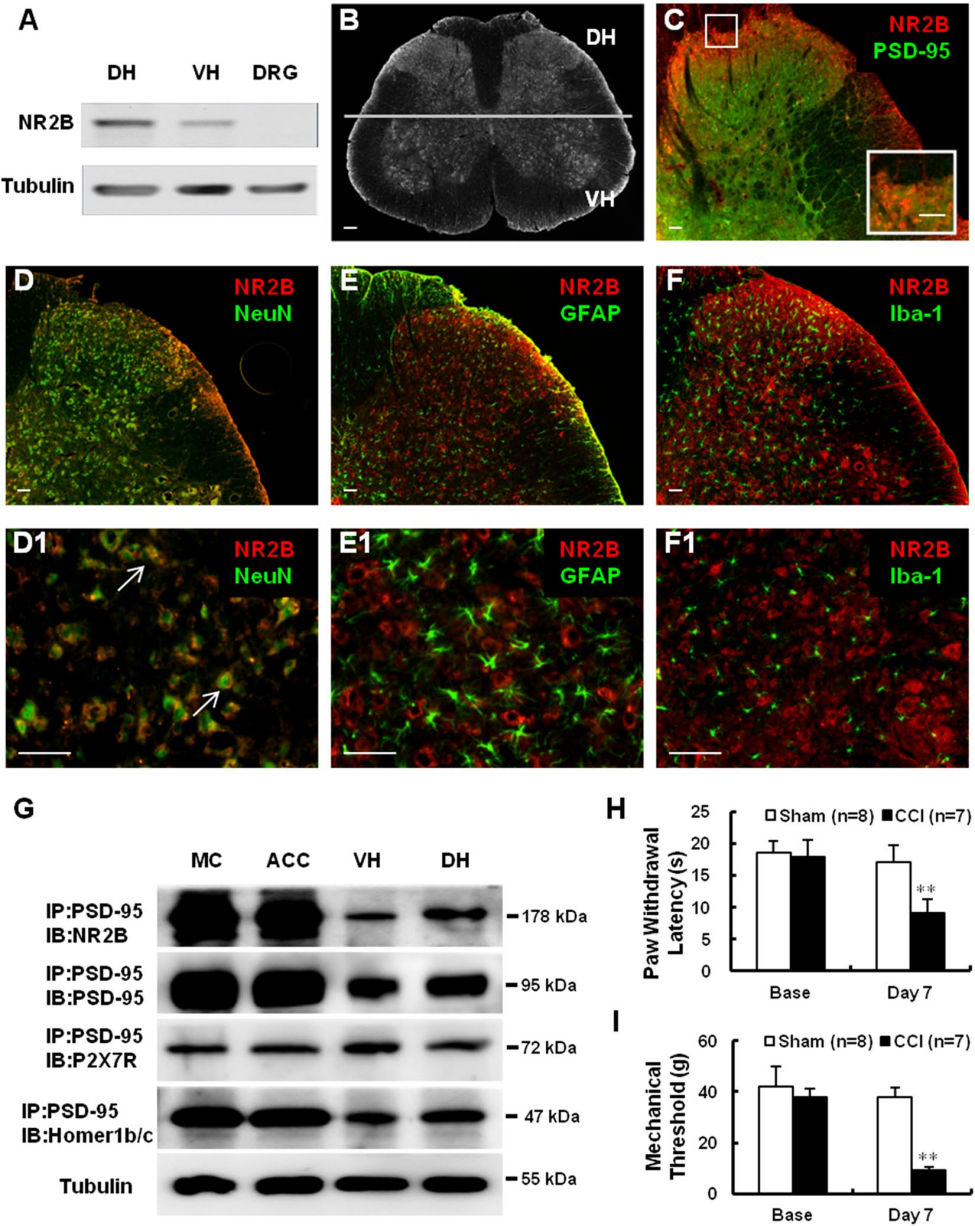
Spinal cord expression of NR2B and PSD-95 and behavioral signs of neuropathic pain induced by CCI (**A**). Western blot showing the expression of NR2B in the DH, VH, and DRG. Tubulin served as the loading control (**B**). Immunohistochemical localization of NR2B in the rat spinal cord. The line depicts dorsal- ventral axis. (**C**–**F1**) Double immunostaining of NR2B (red; **C**–**F1**) with PSD-95 (green; **C**), NeuN (green, a marker of neurons; **D**,**D1**), GFAP (green, a marker of astrocytes; **E**,**E1**), and Iba-1 (green, a marker of microglia; **F**,**F1**). The inset in C shows a magnification of the boxed area; (**D1**,**E1**,**F1**) are enlarged from (**D**–**F**), respectively. Scale bars: (**B**), 100 μm; (**C**–**F1**), 50 μm; inset in (**C**), 50 μm (**G**). Co-immunoprecipitation assays showing PSD-95 interactions with NR2B, P2X7R, and Homer1b/c in different nervous system regions (**H**,**I**). Quantification of thermal paw withdrawal latency (**H**) and paw withdraw mechanical threshold (**I**) 7 days after CCI- or sham-surgery. **P < 0.01 *vs* sham group (*n* = 7–8, Mann-Whitney test). Data are shown as mean ± SEM. CCI, chronic constriction injury; MC, motor cortex; ACC, anterior cingulate cortex; VH, ventral horn; DH, dorsal horn; DRG, dorsal root ganglion; IB, immunoblotting; IP, immunoprecipitation; Base, baseline.

We first examined the area-specific expression of NR2B and its co-expression with PSD-95, in the spinal cord, using immunoblotting (IB) and immunofluorescence. The results showed that the NR2B protein was enriched in the spinal dorsal horn (DH) than the ventral horn (VH); no NR2B signal was detected in the dorsal root ganglion (DRG) of healthy rats (Fig. 1A,B). Double staining of the spinal cord sections for NR2B and PSD-95 revealed that the immunoreactivities of the two proteins co-localized (Fig. 1C). To test whether NR2B was expressed in neurons, microglia, or astrocytes cells, we labeled the sections using antibodies for neuronal nuclear protein (NeuN), ionized calcium binding adaptor molecule 1 (Iba-1), and glial fibrillary acidic protein (GFAP), respectively. The results revealed that NR2B co-localized well with NeuN (Fig. 1D,D1) but showed no overlap with GFAP (Fig. 1E,E1) or Iba-1 (Fig. 1F,F1). This indicates that NR2B is expressed only in neurons and not in DH glial cells and is co-localized with PSD-95.

### Expression and protein interaction of PSD-95 and NR2B in the nervous system

We performed immunoprecipitation (IP) and co-IP assays with PSD-95 precipitates extracted from tissues of normal rats, from different regions of the nervous system, including the motor and anterior cingulate cortex, as well as the DH and VH of the spinal cord. We found that PSD-95 was expressed in these areas and immunoprecipitated with NR2B, the purinergic receptor P2X7 (P2X7R), and Homer1b/c proteins. Moreover, there was an obvious difference in the intensity of protein interaction of PSD-95 with NR2B and Homer1b/c between the cortex and spinal cord. The interaction of PSD-95 and NR2B was much more vigorous in the DH than the VH (Fig. 1G). These findings indicate that in addition to NR2B, PSD-95 interacts with other intracellular signaling molecules (such as Homer1b/c), and cell-surface receptors (such as P2X7).

### CCI-induced behavioral hyperalgesia

In the present study, a model of sciatic nerve CCI was established, in order to mimic clinical chronic neuropathic pain. By using appropriate behavioral paradigms, we found that the paw withdrawal thresholds for either mechanical or thermal stimuli were lower in the CCI than the sham-operated group on day 7, a time-point when the CCI group usually reached the maximal behavioral hyperalgesia after neuropathic-like injury^21^. In addition, compared to pre-operative baseline, both the thermal paw withdrawal latency (**P < 0.01 *vs*. sham, *n* = 7–8; Fig. 1H) and mechanical threshold, of specifically the ipsilateral hindpaw of the CCI group (**P < 0.01 *vs*. Sham, *n* = 7–8; Fig. 1I), were decreased.

### Activation of NR2B/PSD-95/CREB signaling in the spinal DH after CCI

**Figure 2:**
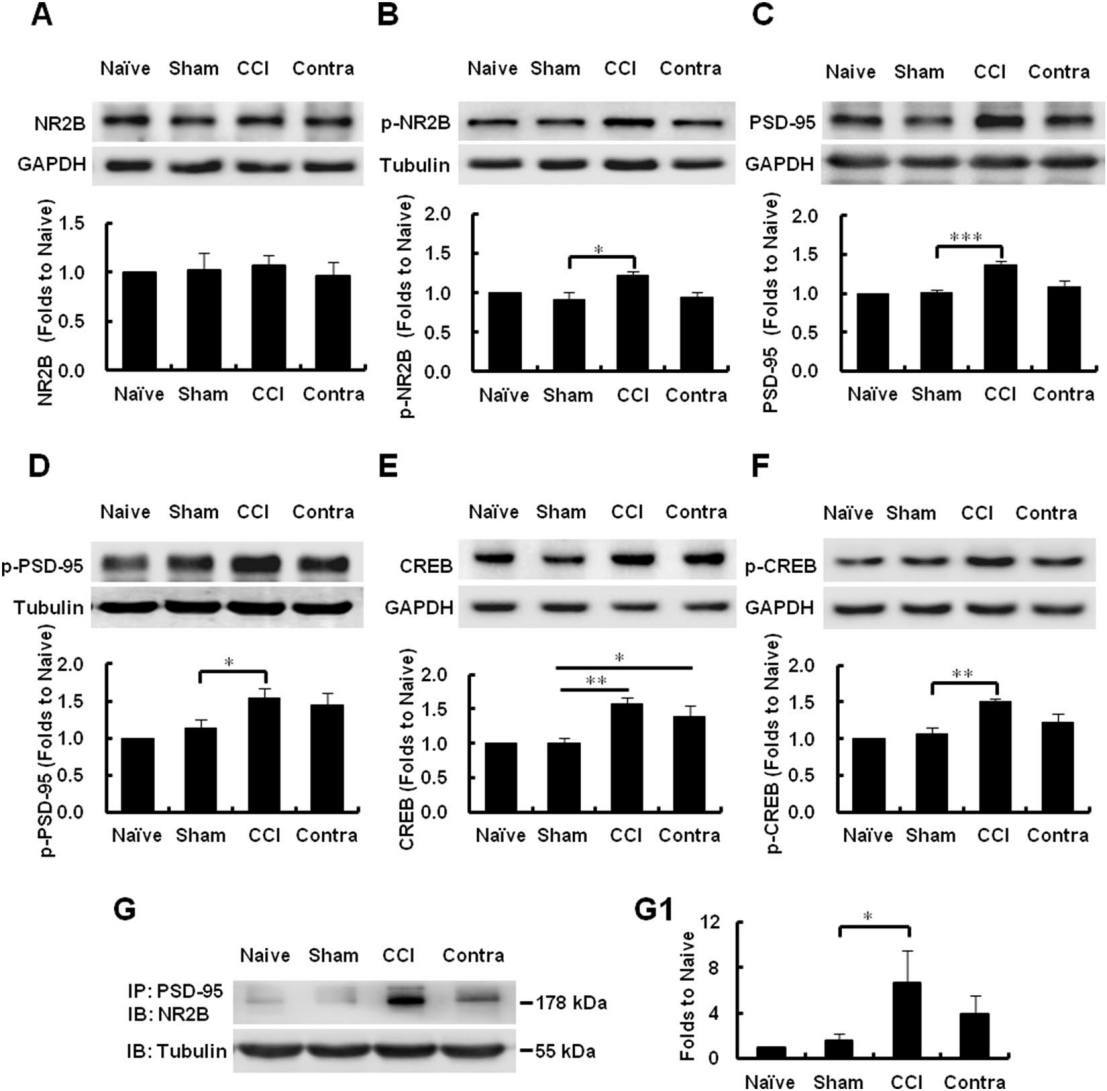
Activation of NR2B/PSD-95/CREB signaling pathway following CCI. (**A**–**F**) Representative uncropped western blots and the respective quantification of the immunoreactive bands, showing protein expression of NR2B (**A**), p-NR2B (**B**), PSD-95 (**C**), p-PSD-95 (**D**), CREB (**E**), and p-CREB (**F**) in the ipsilateral dorsal horn 7 days after CCI or sham operation. Blots were probed with specific antibodies, as indicated. Tubulin or GAPDH were used as loading controls, and run on the same blot. ***P < 0.001, **P < 0.001, *P < 0.05, *n* = 4–6, one-way ANOVA. (**G**,**G1**). Co-immunoprecipitation of PSD-95 with NR2B in the spinal dorsal horn after CCI (**G**) and quantification of the intensity of the immunoreactive bands (**G1**) Note that CCI significantly increases the PSD-95-NR2B interaction (*P = 0.022, *n* = 3–4, Mann-Whitney test). Data are shown as mean ± SEM and represent the fold-change compared to the values of the naïve sample. IB, immunoblotting; IP, immunoprecipitation; CCI, ipsilateral side to chronic constriction injury; Contra, contralateral side to chronic constriction injury.

We further analyzed whether CCI-induced hyperalgesia is associated with the spinal cord expression of NR2B, PSD-95, and CREB, as well as their respective phosphorylated forms. On day 7 after CCI, western blot analyses revealed no significant differences in the overall level of NR2B protein between the CCI- and sham-operated groups (*P* = 0.538, *n* = 5; Fig. 2A). However, the levels of phosphorylated (p) NR2B were higher in the CCI- than the sham-operated animals (*P = 0.04 *vs*. sham, *n* = 4; Fig. 2B). Moreover, CCI provoked the upregulation of PSD-95 (***P < 0.001 *vs*. sham, *n* = 5; Fig. 2C), p-PSD-95 (*P = 0.028 *vs*. Sham, *n* = 5; Fig. 2D), CREB (**P = 0.001 *vs*. sham, *n* = 6; Fig. 2E), and p-CREB (**P = 0.001 *vs*. sham, *n* = 5; Fig. 2F) in the DH, evident by the significant increase in the bands’ intensity.

Next, to study whether the NR2B-PSD-95 interaction is involved in the generation and maintenance of neuropathic pain, we performed co-IP analysis in samples from sham- and CCI-operated animals. We found that the amount of PSD-95-bound NR2B, in the ipsilateral DH, was higher in the CCI than in the control group (*P = 0.022 *vs*. sham, *n* = 3–4; Fig. 2G,G1). Taken together, these data indicate that CCI induces the accumulation and activation of NR2B/PSD-95/CREB signaling pathway in the spinal cord, as well as the physical interaction between PSD-95 and NR2B in the spinal DH.

### Effects of intrathecal Ro 25-6981 injection on mechanical and thermal hyperalgesia

**Figure 3:**
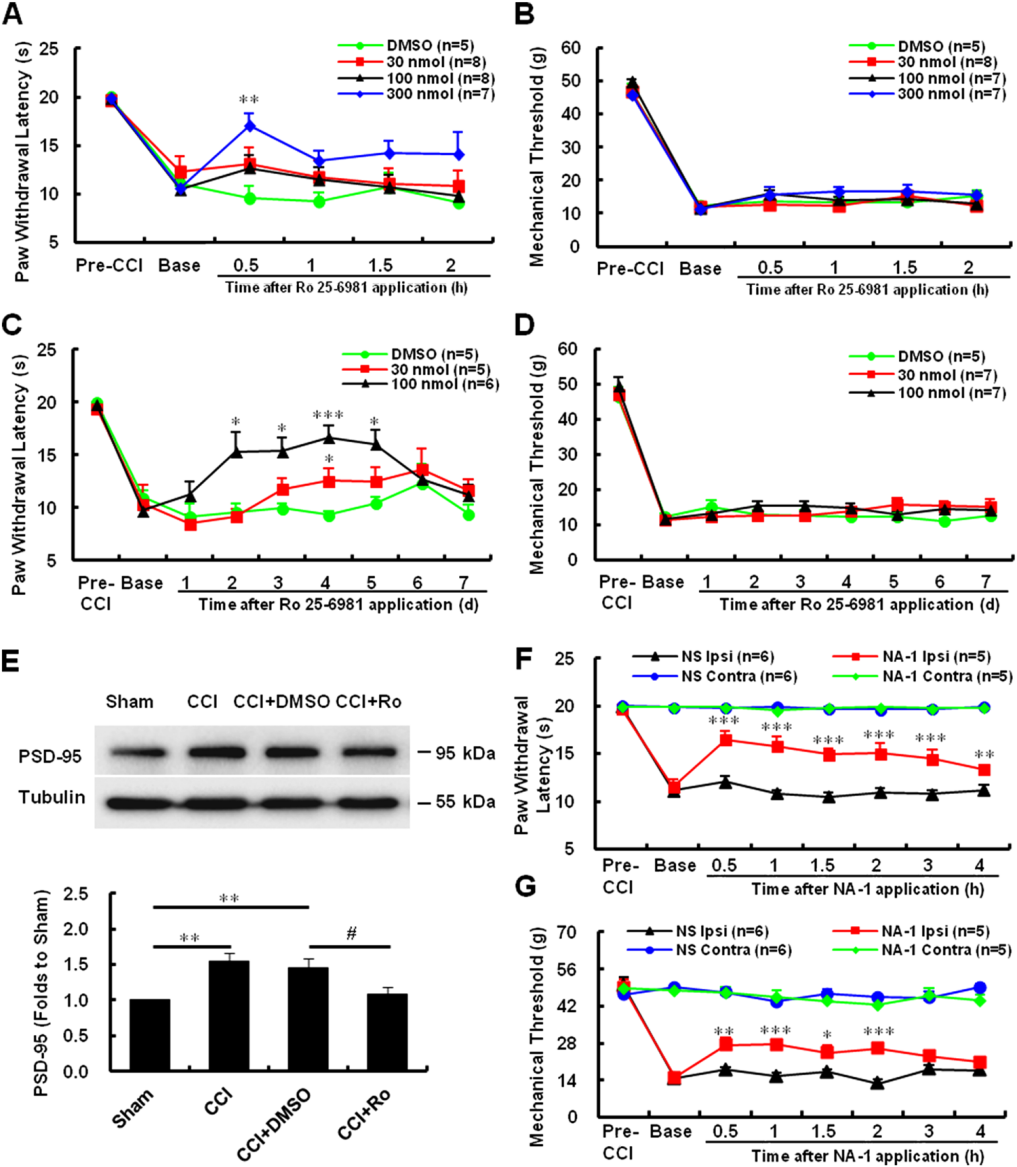
Effect of Ro 25-6981 and NA-1 on paw withdrawal responses to noxious stimuli and on the expression of PSD-95 (**A**–**D**). Quantification of thermal paw withdrawal latency (**A**,**C**) and paw withdrawal mechanical threshold (**B**,**D**) at different time-points after injection of DMSO or different concentrations of Ro 25-6981, as indicated. (***P < 0.001, **P < 0.01, *P < 0.05 *vs*. DMSO, *n* = 5–8, two-way ANOVA) (**E**). Representative uncropped western blots and quantification of the immunoreactive bands showing the effects of Ro 25-6981 on PSD-95 expression after CCI surgery. Tubulin served as the loading control and run on the same blot. The data are shown as the mean ± SEM and represent the fold-change compared to sham-operated mice. **P < 0.01 *vs*. sham, ^#^*P* = 0.013 *vs*. CCI + DMSO, *n* = 5, one-way ANOVA (**F,G)**. Quantification of thermal paw withdrawal latency (**F**) and paw withdrawal mechanical threshold (**G**) at different time-points after injection of NA-1 (125 ng/10 μL) 7 days after CCI surgery (*P < 0.05; **P < 0.01; ***P < 0.001 *vs*. NS ipsilateral, *n* = 5-6, two-way ANOVA). The data are shown as the mean ± SEM. CCI, chronic constriction injury; NS, vehicle; Ipsi, ipsilateral; Contra, contralateral; Ro, Ro 25-6981.

To further explore the role of NR2B in CCI-associated neuropathic pain, in regard to mechanical and thermal hyperalgesia, we intrathecally injected Ro 25-6981 (30, 100, or 300 nmol, in 10 μL volume), or DMSO, 7 days after CCI. Behavioral tests in responses to mechanical or thermal stimuli were performed before and after the injection (Fig. 3A–D). To examine acute anti-nociception, testing was conducted at four time-points (0.5, 1, 1.5, and 2 h) after the application. We found that intrathecal administration of Ro 25-6981 attenuated CCI-induced thermal hyperalgesia, while vehicle injection had no significant effects. This robust reversal of thermal hyperalgesia peaked at 30 min after Ro 25-6981 (300 nmol/10 μL) injection (**P < 0.01 *vs*. DMSO, *n* = 5–8, Fig. 3A). For chronic anti-nociception analysis, the behavioral testing was conducted at 120 min after drug administration for 5 consecutive days. Our results showed that Ro 25-6981 (100 nmol/10 μL) markedly reduced thermal hyperalgesia, in a time-dependent manner, from the 2nd to the 5th day of administration, compared to the vehicle group (*P < 0.05; ***P < 0.001 *vs*. DMSO, *n* = 5–6; Fig. 3C). In the CCI group, drug withdrawal at days 6 and 7 caused a slight decrease in the paw withdrawal latency compared to day 5. The different doses of Ro 25-6981 did not produce any significant analgesic effect on mechanical hyperalgesia at the same time-points (Fig. 3B,D).

### Effects of intrathecal Ro 25-6981 application on the expression of PSD-95

Next, we applied 100 nmol/10 μL of Ro 25-6981 and detected the expression of PSD-95 protein by western blotting. We found that intrathecal injection of Ro 25-6981, but not dimethylsulfoxide (DMSO), 7 days after CCI surgery, significantly decreased the CCI-induced upregulation of PSD-95 in the spinal lumbar DH segments (**P < 0.01 *vs*. sham, ^#^*P* < 0.05 *vs*. CCI + DMSO, *n* = 5; Fig. 3E), suggesting that Ro 25-6981 inhibits PSD-95 expression in the spinal DH.

### Effects of intrathecal NA-1 injection on mechanical and thermal hyperalgesia

To confirm the role of PSD-95 in CCI-associated neuropathic pain, we intrathecally injected the PSD-95 antagonist NA-1 (125 ng in 10 μL) 7 days after CCI. Rats were randomly assigned to the NA-1 (*n* = 5) or control (normal saline, NS; *n* = 6) groups. Behavioral tests for assessing responses to mechanical or thermal stimuli were performed before and at six time-points (0.5, 1, 1.5, 2, 3, and 4 h) after NS or NA-1 intrathecal injection. Treatment with NA-1 produced a reversal of both the CCI-induced thermal (***P < 0.001; **P < 0.01 *vs*. NS Ipsi, *n* = 5–6; Fig. 3F) and mechanical (***P < 0.001; **P < 0.01; *P < 0.05 *vs*. NS Ipsi, *n* = 5-6; Fig. 3G) hyperalgesia of the ipsilateral hindpaw. These differences were statistically significant throughout the 4-hour testing period. Thus, intrathecal NA-1 attenuated CCI-induced thermal and mechanical hypersensitivity, which suggests that activation of PSD-95 is required for the maintenance of CCI-induced neuropathic pain.

### Intrathecal Ro 25-6981 or NA-1 administration does not produce sedation

All rats treated with Ro 25-6981 or NA-1 had a score of 0 on both the posture and righting reflex scales, indicating that Ro 25-6981 or NA-1 do not induce sedation. In addition, none of the rats, treated with either drug, showed any other adverse effects, including motor deficits or body weight abnormalities (data not shown).

## Discussion

In the present study, we demonstrated that CCI-induced neuropathic pain is associated with the up-regulation of total and p-PSD-95 in the spinal cord. At the same time as reducing abnormal thermal pain-related behaviors by perturbing the NR2B function, we showed that Ro 25-6981 inhibits PSD-95 upregulation in the spinal cord. Furthermore, we found that the inhibition of PSD-95 by its antagonist NA-1, also attenuates the behavioral hypersensitivity induced by CCI. Given that coupling of PSD-95 to NR2B might facilitate downstream NMDAR signaling, the present study suggests that the upregulation of spinal cord PSD-95 might play a critical role in the manifestation of central sensitization and development of chronic pain.

PSD-95 is well known to play an essential role in regulating the interactions between glutamate receptors and downstream signaling molecules. NMDARs, in combination with members of the membrane-associated guanylate kinase (MAGUK) scaffolding protein family, constitute essential components of post-synaptic macromolecular signaling complexes, which serve to propagate glutamate responses intracellularly^22^. The present study confirmed the basis of PSD-95 function, by demonstrating that PSD-95 binds to NR2B, P2X7R, and Homer1b/c proteins, *in vivo*, in different regions of the nervous system. These results are consistent with previous studies showing that PSD-95 is involved in synaptic targeting and interacts with the majority of intracellular signaling molecules and receptors^18,23,24^. However, our results extend the interaction profile of PSD-95, which seems to include new signaling proteins and cell-surface receptors.

Chronic primary afferent stimulation, induced by CCI, causes NR2B-related, NMDAR-mediated plastic changes in spinal neuronal activity, producing central sensitization of DH neurons^25^. Wilson *et al*.^16^ showed that spinal cord NR2B expression increases in a model of injury, similar to CCI; in contrast, we demonstrated that CCI does not affect spinal cord NR2B expression, in accordance with other reports^17–20^. These results suggest that pain transmission signals, induced by CCI, are not exerted via NR2B protein level alterations. Interestingly, we showed that phosphorylation of NR2B (p-NR2B, Ser1303) is upregulated following CCI, in line with previous studies on the involvement of p-NR2B (Tyr1472) in the development of inflammatory^17^ and neuropathic pain^26^, in the DH. In addition, several lines of evidence support the notion that spinal NR2B phosphorylation plays a crucial role in the development of chronic visceral pain, which is mediated by spinal NR2B and tyrosine kinase^27,28^. Furthermore, we found a dramatic upregulation of PSD-95 expression following CCI, contrary to previous studies, in which PSD-95 expression did not change after spinal nerve ligation (SNL)^18,23^ or complete Freund’s adjuvant injection^29^. The discrepancy between ours and previous findings may be attributed to the different animal species or models used to evaluate experimental chronic pain. Further research is warranted to elucidate the relevant underlying mechanisms and the clinical significance of these findings.

We also explored the role of PSD-95-NR2B interaction in chronic pain, in our CCI model. Our findings showed that the interaction between the two proteins is enhanced upon injury and paralleled with the induced hyperalgesia, indicating that NR2B and PSD-95 are associated with the development of neuropathic pain. This is in line with previous reports, which have shown that NR2B effects in pain are alleviated by Ro 25-6981 administration, which has analgesic effects^17,23^. In the present study, we also confirmed that Ro 25-6981 exerts an analgesic effect in the CCI model. Our behavioral analyses showed that spinal application of Ro 25-6981 attenuates thermal hyperalgesia without affecting mechanical hypersensitivity induced by CCI. The findings are consistent with those of a previous report showing that the NMDA receptor antagonist memantine produces therapeutic effects on thermal hyperalgesia in the CCI model^30^. However, application of another NR2B antagonist, ifenprodil, prior to nerve injury, attenuates mechanical allodynia but not thermal hyperalgesia^31^. On the contrary, D’Mello *et al*.^11^ found that perturbing NR2B/PSD-95 interaction, by using Tat-NR2B9c, attenuates cold and mechanical allodynia in an SNL model. These discrepancies may result from the different animal models used in these studies. Another reason might be the instrument used for measurements in the present study, which exhibits rather low sensitivity compared to the conventional von Frey filaments, according to our experience. The third reason may be the different agent used in the different studies. Therefore, further investigations are required to address whether different NR2B antagonists and different neuropathic models have similar effect profiles and whether specific NR2B antagonists are selective for thermal or mechanical hyperalgesia. In addition to antagonist treatment, several studies have used genetic or pharmacological methods to perturb the NR2B/PSD-95 interaction and have found that this also leads to analgesia^18,19,23,32^.

Furthermore, we revealed that Ro 25-6981 administration decreases PSD-95 levels, after CCI, further suggesting that the function of NR2B is mediated by PSD-95. The reciprocal experiment involving the spinal application of the PSD-95 inhibitor NA-1, showed similar results, i.e., attenuation of the behavioral hypersensitivity induced by CCI. A previous study reported that disruption of PSD-95-NMDAR interactions, with the Tat-NR2B9c peptide, enhances Ca^2+^/CREB-mediated neuroprotection^33^. The authors found that the Tat-NR2B9c neuroprotective effect against oxygen-glucose deprivation and NMDA toxicity occurs in parallel with the activation of calmodulin kinase signaling, depending on the sustained phosphorylation of CREB and its activator Calcium/calmodulin-dependent protein kinase type IV (CaMKIV)^33^. CREB is a constitutively expressed transcription factor and has multiple functions in the nervous system, including synaptic plasticity and nociceptive processing. CREB and p-CREB (Ser133) have been involved in the development of neuropathic^34^ and inflammatory pain^35^. The present study confirmed that CREB is activated following CCI. These results are consistent with several previous studies^34,36^, indicating that p-CREB is an important modulator of neuropathic pain pathogenesis. Since CREB is a critical element in modulating gene expression, it is reasonable that CREB might play roles in PSD-95 and NR2B expression/phosphorylation, and thus involved in PSD-95/NR2B pathway activation in response to CCI. However, the exact mechanism underlying CREB modulation of PSD-95/NR2B activation needs to be further investigated.

## Conclusions

Our study demonstrated that activation of NR2B/PSD-95/CREB signaling in neuropathic pain is a characteristic of pain hypersensitivity, while NR2B blockade attenuates CCI-induced thermal hyperalgesia, as well as the upregulation of PSD-95. Overall, our results suggest that chronic anti-nociception, produced by the NR2B antagonist, might occur via the inhibition of spinal cord PSD-95 upregulation. Thus, our study provides insight on NR2B signaling as a target for the treatment of neuropathic pain.

## Materials and Methods

### Animal handling

Adult male Wistar rats (220–280 g) were provided by the Animal Center of the Chinese Academy of Sciences (Shanghai, P. R. China). Rats were housed in a 12-h day and night cycle with free access to water and food pellets. Rats were acclimatized to the housing facility for 3 days before starting the experiments. The experimental protocol was performed with the approval of the Animal Care Committee at Zhejiang University and according to the ethical guidelines for investigation of experimental pain in animals^37^. Utmost efforts were made to minimize the number and distress of the animals used.

### CCI-induced neuropathic pain

The CCI surgical procedure was performed as previously described by Bennett and Xie^38^. Briefly, rats were anesthetized by inhalation of isoflurane, and the left sciatic nerve was exposed and freed from the underlying connective tissues. Three ligations were placed around the nerve with 4-0 chromic gut sutures, and the atypical twitch of the hind paw confirmed the nerve constriction. For the sham-operated group of animals, the identical surgical procedure was carried out, but without the ligation of the sciatic nerve. All animals received an antibiotic injection (penicillin 0.5 mL, 160,000 U/mL, subcutaneously) to prevent infection.

### Intrathecal catheterization

A polyethylene-10 catheter (Smiths Medical, UK) was implanted into the subarachnoid space, in line with the spinal lumbar enlargement, as described by Storkson *et al*.^39^. The catheter was then capped and immobilized onto the skin after closure of the incision. Rats showing any neurological deficits after surgery were excluded from the study. Each subject was then injected with 10 μL of Ro 25-6981 (diluted in DMSO), NA-1 (diluted in NS), or the respective vehicle (DMSO or NS) as control, followed by a flush of 10 μL NS to fill in the dead space (6–7 μL).

### Behavioral testing

In order to determine paw withdrawal threshold upon a mechanical stimulus, animals were placed in a cage with a wire meshed floor and tested using an electronic von Frey Anesthesiometer (Model 2390, IITC/life Science, Victory Blvd Woodland Hills, CA) with a flexible probe, as described in our previous report^40^. Three measurements were made per animal, and the average value was obtained. Hind-paw withdrawal latency to noxious thermal stimuli was measured with an apparatus (Model 336, IITC/life Science, Victory Blvd Woodland Hills, CA) described by Hargreaves *et al*.^41^. Rats were placed in a Plexiglas chamber with a glass floor, above a light box. Heat was applied by aiming a beam of light from the light box to the hind paw. The delay of foot lift after application of the light beam was defined as the thermal paw withdrawal latency. Each trial was repeated three times, with 5-min intervals and a cut-off time of 20 s, to avoid possible tissue damage. Sedation and motor function were tested immediately after pain-related behavioral testing, based on a 5-point scale of righting reflexes (0 = struggle, 4 = no movement) and posture (0 = normal, 4 = flaccid atonia)^42,43^.

### Co-IP analysis

The spinal lumbar DH was isolated, homogenized in ice-cold lysis buffer (Beyotime Institute of Biotechnology, China), and centrifuged at 5000 rpm for 15 min at 4 °C. The protein concentration in the supernatant was determined by the bicinchoninic acid assay. The supernatants were then incubated with 2 μg mouse monoclonal PSD-95 antibody (Santa Cruz Biotechnology, Inc., Santa Cruz, CA) overnight at 4 °C. Then, 30 μL of protein G magnetic beads (Millipore Bioscience, MA) were added to the reaction and incubated for an additional 2 h. Samples were centrifuged at 2500 rpm for 5 min and the pellets were washed three times in lysis buffer. Bound proteins were extracted by 1× sodium dodecyl sulfate-polyacrylamide gel electrophoresis sample buffer (10 μL), boiled at 100 °C for 5 min, and then centrifuged at 5,000 rpm for 10 s. The supernatants were subsequently analyzed by immunoblotting according to standard procedures described below.

### IB analysis

IB was performed as described previously^35^. Proteins were separated on a 10% gel and transferred onto a polyvinylidene difluoride membrane (Thermo Fisher Scientific Inc., MA) using a wet transfer apparatus (Bio-Rad Laboratories Inc., CA). Each membrane was blocked for 1 h at room temperature and then incubated with primary antibodies against PSD-95 (1:200; Santa Cruz Biotechnology Inc., CA), NR2B (1:2000; Proteintech, Chicago, IL), p-NR2B (Ser1303, 1:1000; Abcam, UK), PSD-95 (1:2000; Abcam), p-PSD-95 (Ser295, 1:5000; Abcam), CREB (1:500; Cell Signaling Technology, MA), p-CREB (Ser133, 1:2000; Millipore, CA), tubulin (1:2000; Beyotime Institute of Biotechnology), or GAPDH (1:10000; Aksomics Inc., China) overnight at 4 °C. The blots were then washed and incubated with a peroxide-conjugated secondary antibody (1:2000; Beyotime Institute of Biotechnology) for 2 h at room temperature. Protein bands were visualized by chemiluminescence using an enhanced chemiluminescence reagent (Thermo Fisher Scientific Inc.) and captured using a ChemiDoc MP System (Bio-Rad Laboratories Inc., CA). Tubulin was used as a loading control. Commercial markers (Thermo Fisher Scientific Inc.) were used as molecular weight standards.

### Immunofluorescence

Rats were anesthetized and perfused through the ascending aorta with NS, followed by 300 mL of 4% paraformaldehyde in 0.1 M phosphate buffer. Lumbar enlargement segments were then harvested, postfixed at 4 °C overnight, and dehydrated in 30% sucrose. Afterwards, 30-μm free-floating transverse sections were cut at −20 °C in a freezing microtome. Sections were collected in phosphate-buffered saline and double-labeled to investigate the co-expression of NR2B and neuronal, glial, or microglial markers. Specifically, spinal sections were incubated for 48 h at 4 °C in a mixture of mouse anti-NR2B (1:200; Abcam) and rabbit anti-PSD-95 (1:2000; Abcam), rabbit anti-NeuN (1:500; Abcam), rabbit anti-GFAP (1:500; Proteintech, IL), or rabbit anti-Iba-1 (1:1000; Abcam) antibodies. The sections were then incubated with a mixture of Cy3-conjugated anti-mouse IgG (1:200; Proteintech) and fluorescein isothiocyanate-conjugated anti-rabbit IgG (1:200; Proteintech), for 2 h at room temperature. Fluorescent signal was viewed with a fluorescence microscope (OLYMPUS, x-Cite 120, Japan) with the appropriate filters.

### Drug application

The selective NR2B antagonist Ro 25-6981 was purchased from Sigma-Aldrich (St-Louis, MO), and dissolved in 100% DMSO. Animals were randomly divided into two groups; the vehicle and Ro 25-6981 groups. Different amounts of Ro 25-6981 (30, 100, or 300 nmol) were intrathecally injected in a volume of 10 μL. To examine acute anti-nociception, testing was conducted at four time-points (0.5, 1, 1.5, and 2 h) after the application. For chronic anti-nociception analysis, the behavioral testing was conducted at 2 h after drug administration for 5 consecutive days. The PSD-95 inhibitor NA-1 (Tat-NR2B9c) was supplied by MedChem Express (MCE, NJ), and administered intrathecally at a single dose of 125 ng in 10 μL saline. A vehicle solution of 10 μL saline was administered in the control group. Behavioral testing was performed before and after drug administration, at six time-points (0.5, 1, 1.5, 2, 3, and 4 h) after vehicle (NS) or NA-1 intrathecal injection. During the experimental period, we did not observe any adverse events, including weight loss, diarrhea, or obvious motor deficiency resulting from the daily Ro 25-6981 or NA-1 administration.

### Statistical Analysis

Data are presented as mean ± standard error of the mean (SEM). The independent Student’s *t*-test was used to analyze the effectiveness of the CCI model. The paw withdrawal threshold and protein abundance were analyzed by one- or two-way analysis of variance (ANOVA) followed by the least significant difference test for multiple comparisons. Non-parametric analyses were performed using the Mann-Whitney test. Results were considered to be statistically significant if *P* < 0.05.
